# Universal growth of ultra-thin III–V semiconductor single crystals

**DOI:** 10.1038/s41467-020-17693-5

**Published:** 2020-08-07

**Authors:** Yunxu Chen, Jinxin Liu, Mengqi Zeng, Fangyun Lu, Tianrui Lv, Yuan Chang, Haihui Lan, Bin Wei, Rong Sun, Junfeng Gao, Zhongchang Wang, Lei Fu

**Affiliations:** 1 College of Chemistry and Molecular Sciences, Wuhan University, Wuhan, 430072 China; 2 Laboratory of Materials Modification by Laser, Ion and Electron Beams, Dalian University of Technology, Ministry of Education, Dalian, 116024 China; 3 International Iberian Nanotechnology Laboratory (INL), Av. Mestre Jose Veiga s/n, 4715-330 Braga, Portugal

**Keywords:** Two-dimensional materials, Electronic properties and materials

## Abstract

Ultra-thin III–V semiconductors, which exhibit intriguing characteristics, such as two-dimensional (2D) electron gas, enhanced electron–hole interaction strength, and strongly polarized light emission, have always been anticipated in future electronics. However, their inherent strong covalent bonding in three dimensions hinders the layer-by-layer exfoliation, and even worse, impedes the 2D anisotropic growth. The synthesis of desirable ultra-thin III–V semiconductors is hence still in its infancy. Here we report the growth of a majority of ultra-thin III–V single crystals, ranging from ultra-narrow to wide bandgap semiconductors, through enhancing the interfacial interaction between the III–V crystals and the growth substrates to proceed the 2D layer-by-layer growth mode. The resultant ultra-thin single crystals exhibit fascinating properties of phonon frequency variation, bandgap shift, and giant second harmonic generation. Our strategy can provide an inspiration for synthesizing unexpected ultra-thin non-layered systems and also drive exploration of III–V semiconductor-based electronics.

## Abstract (web summary)

Here, the authors synthesize a variety of ultra-thin III–V single crystals, ranging from ultra-narrow to wide bandgap semiconductors, through enhancing the interfacial interaction between the III–V crystals and the growth substrates.

## Introduction

III–V semiconductors have profound underlying physics, such as high charge carrier mobility^1^, high electron drift velocity^2^, efficient light detection^3^ and emission^4^, which endow them great potential in high-speed, high-frequency electronics^1^ and high-efficiency optoelectronics including solar cells^5^, lasers^6^, and light-emitting diodes^4,7^. Once it comes to two dimensions, enormous unique characteristics emerge, such as two-dimensional (2D) electron gas^8^, enlarged lattice constant^9,10^, blue-shifted bandgap^11,12^, strong electron–hole interaction, and strongly polarized light emission favoring the nonlinear optics^13^, which stimulate the interest of their property exploration^14^ in the 2D limit.

However, the strong covalent bonding in the inherent zinc blende (ZB)^15,16^ or wurtzite structures^17^ of these non-layered III–V crystals hinders the layer-by-layer exfoliation, and even worse, it greatly impedes the 2D anisotropic growth. Therefore, the synthesis of desirable ultra-thin III–V compound semiconductors is still in its infancy. Currently, among the various semiconductors crystals available, only ultra-thin GaN crystals have been synthesized^11,12,18^. The quantum confinement induced characteristics of a majority of these compounds, such as phosphides and antimonides, remain unproven in the 2D limit. The root cause is that these growth methods rely heavily on a nanoscale confined space^12^ or a predesigned template^11,18^ with harsh requirements, thus seriously restricting the expansion of the growth methods to other ultra-thin III–V systems. Therefore, it is urgent to develop a general approach without strict presetting for the efficient growth of these ultra-thin III–V systems.

Here, alloy substrates with improved adhesion strength toward III–V crystals were designed to enhance the interfacial interaction between the substrates and the materials. Thus, the resulted surface energy difference can trigger the 2D layer-by-layer (LBL) growth behavior of III–V crystals on the substrates, further providing a driving force to stimulate the 2D anisotropic growth of various representative non-layered III–V single crystals ranging from ultra-narrow to wide bandgap semiconductors. These highly-crystalline ultra-thin single crystals exhibit intriguing properties of strong phonon confinement, apparent bandgap shift, and giant second harmonic generation (SHG). These as-synthesized ultra-thin III–V systems can provide a fertile platform for probing their intrinsic physics. The as-proposed method offers a universal strategy for adjusting the growth mode of the materials and paves a route to achieve 2D crystals, which can bring forth inspiration for realizing the anisotropic growth of unexpected 2D materials, especially for the non-layered systems.

## Results

### Universality of the growth strategy

**Fig. 1:**
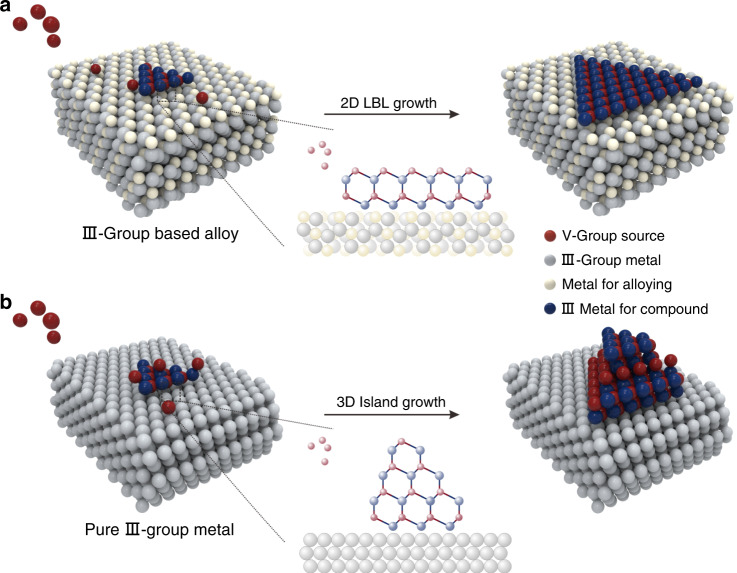
Schematic illustration of the growth process of III–V crystals. **a** The schematic of the 2D LBL growth process of ultra-thin III–V single crystals on an alloy substrate. **b** The schematic of the 3D island growth process of bulk III–V single crystals on the pure III-group metal substrate.

To create the surface energy differences, III-group-metal based alloys (e.g., AuIn_2_) with enhanced adhesion strength toward III–V crystals were employed as growth substrates. The strengthened interfacial interaction enables the growth of III–V crystals to proceed in the 2D LBL growth mode on the substrates, further leading to the formation of ultra-thin non-layered III–V single crystals, as sketched in Fig. 1a. However, in terms of the growth process performed on a pure III-group metal substrate with a weak adhesion strength (Fig. 1b), which leads to a three-dimensional (3D) island growth mode of the III–V crystals. An isotropic growth behavior would be triggered, thus resulting in the formation of bulk crystals.

**Fig. 2:**
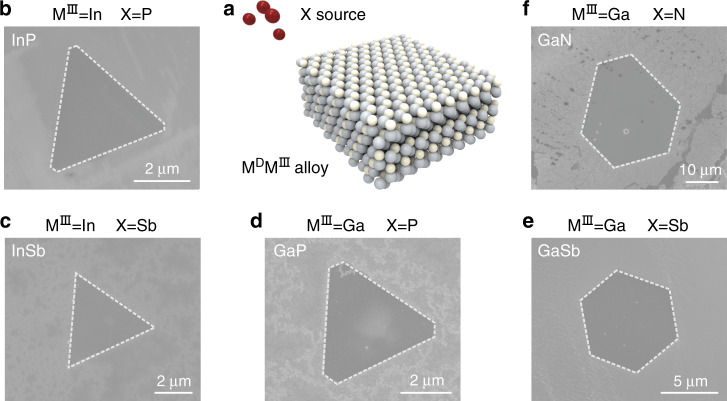
Universality of the LBL growth toward ultra-thin III–V single crystals. **a** The schematic diagram of the growth process. **b**–**f** SEM images of the synthesized InP, InSb, GaP, GaSb, and GaN single crystals, respectively.

Representative ultra-thin III–V single crystals were synthesized via the LBL growth strategy (Fig. 2a). Au is added to the In metal to form AuIn_2_ substrate and red P powders are employed as V-group precursors during the chemical vapor deposition (CVD) process, leading to the growth of ultra-thin InP, as revealed in the scanning electron microscope (SEM) image (Fig. 2b). The typical Raman spectrum of InP (Supplementary Fig. 1a) characterizes two peaks at 304 cm^–1^ and at 342 cm^–1^, which are assigned to the transverse optical (TO) mode and the longitudinal optical (LO) mode, respectively. The high intensity of the TO phonon frequency is deemed to be correlated with a polarized first-order Raman scattering originated from the interplay of phonon confinement^19^. This spectral polarization confirms that the ultra-thin InP crystals are of high quality. Compared with the Raman spectrum of the bulk InP where the two peaks are located at 306 and 345 cm^–1^ (Supplementary Fig. 2), the TO and LO phonon frequencies of ultra-thin InP exhibit distinguishable downshifts, which can be attributed to the size-induced phonon confinement effect^20,21^ and the increased lattice constant^22^ owing to the reduced thickness.

We further show that varying the V-group precursors to Sb gives rise to the successful growth of ultra-thin InSb single crystals (Fig. 2c) on the AuIn_2_ substrate. The TO (177 cm^−1^) and LO (187 cm^−1^) phonon frequencies (Supplementary Fig. 1b) of InSb single crystals exhibit similar peak-position-shift phenomena observed in the ultra-thin InP single crystals, which stems from the phonon confinement as well^23^. When varying the alloy substrate to be AuGa, ultra-thin GaP (Fig. 2d) and GaSb (Fig. 2e) single crystals can be synthesized. Compared with the Raman peaks of bulk GaP^24^ and GaSb^23^, the peaks of the ultra-thin GaP (Supplementary Fig. 1c) and GaSb (Supplementary Fig. 1d) also exhibit obvious downshifts. Apart from the narrow-bandgap III–V compounds mentioned above, the wide bandgap compound, such as ultra-thin GaN crystal (Fig. 2f), can also be synthesized on the AuGa substrates once the precursors are changed to urea ((NH_2_)_2_CO) powders. The strong peak at 566 cm^–1^ (E_2_) suggests the high quality of ultra-thin GaN^11^. The growth of these representative III–V semiconductors provides a reliable evidence for the great universality of our strategy.

### Theoretical analysis of the LBL growth process

The fundamental thermodynamic process of the growth was then explored for in-depth comprehending of how the InP growth modes were affected by the substrate with a strong adhesion strength. According to the classical thin film growth theory^25^, the growth modes of the material on a substrate can be defined by the difference of surface energies (Δ*γ*), which can be expressed as

1 $${\Delta}\gamma = \gamma _m + \gamma _i-\gamma _s$$

where the *γ*_*m*_ is the surface energy of the material, *γ*_*s*_ is the surface energy of the substrate, and *γ*_*i*_ is the interface energy between the material and the substrate. In addition, according to the Dupré equation

2 $$\gamma _i = \gamma _m + \gamma _s-\beta _i$$

where the *β*_*i*_ is the adhesion energy between the material and the substrate, the Δ*γ* can then be described as^26^

3 $${\Delta}\gamma = 2\gamma _m-\beta _i$$

if Δ*γ* < 0, the material growth can proceed in the 2D LBL growth mode on the substrate, which signifies the requirements of a high adhesion energy between the material and the substrate.

**Fig. 3:**
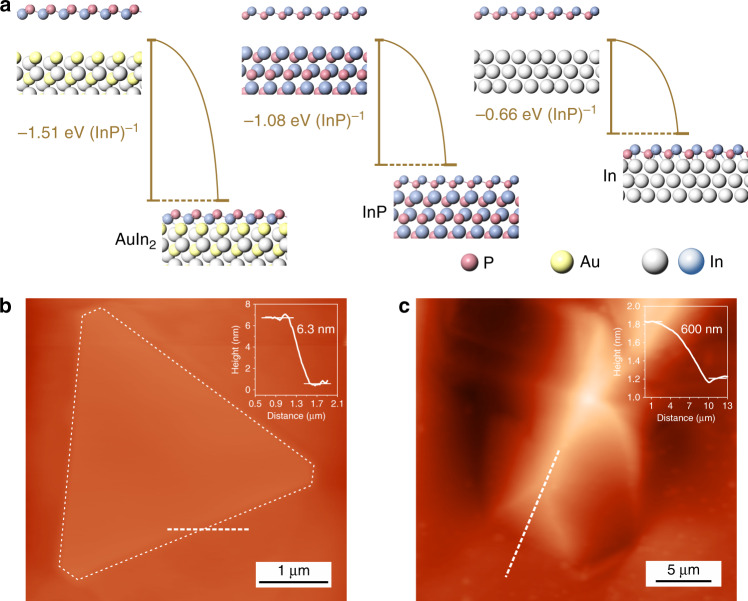
Theoretical analysis of the growth of InP. **a** The adhesion energies of InP layers on the surfaces of AuIn_2_, InP, and In substrates. **b** The AFM image of a typical ultra-thin InP single crystal grown on AuIn_2_. The inset shows the corresponding height profile. **c** The AFM image of a bulk InP crystal grown on In. The inset shows the corresponding height profile.

Given this, enhancing the interaction between the III–V compounds with the substrate can promote the LBL growth behavior and enable us to achieve the ultra-thin III–V single crystal. Here, the adhesion strengths of the growth substrates were adjusted via constructing appropriate III-metal-based alloy substrates. Taken the growth of ultra-thin InP as an example, we performed the density functional theory (DFT) calculations to investigate the interface adhesion energies of InP crystal layer on various substrate surfaces. As shown in Fig. 3a, the results suggest the adhesion energy of InP layer on the AuIn_2_ alloy is about 1.51 eV (InP)^–1^, where (InP)^–1^ indicates per unit cell of InP crystal. The value is much larger than that on the InP substrate of 1.08 eV (InP)^–1^, while the adhesion energy on the In metal exhibits the weakest value of 0.66 eV (InP)^–1^. Here, the formation of the AuIn_2_ alloy is confirmed by the X-ray diffraction (XRD) spectra (Supplementary Fig. 3) and the X-ray photoelectron spectra (XPS) (Supplementary Fig. 4). Regarding that the surface energy of the (111) plane of InP is ~0.66 eV (InP)^–1^ in the reported work^27^, the growth of InP on the AuIn_2_ substrate would prefer to proceed in a 2D LBL growth mode as the Δ*γ* = –0.19 eV (InP)^–1^ is <0. In addition, the Δ*γ* of the InP–In system was calculated as a positive value of 0.66 eV (InP)^–1^, which indicates a 3D island growth behavior of the InP crystals and the formation of bulk crystals on the In surface.

The atomic force microscopy (AFM) image of the typical InP crystal grown on the AuIn_2_ alloy with high adhesion strength (Fig. 3b) suggests a triangular morphology with a flat surface. The corresponding height profile (inset of Fig. 3b) indicates an ultra-thin thickness of ~6.3 nm, confirming the ultra-thin characteristic of the InP crystal grown on the AuIn_2_ alloy. The AFM image of the crystals grown on the In metal is presented in Fig. 3c, where a height value of ~600 nm is observed. The result signifies a bulk InP crystal grown on the substrate with a weak adhesion strength, thus validating our growth mechanism.

Here, we believe that the enhanced interaction between the InP layer and AuIn_2_ substrate is originated from the high adsorption strength of AuIn_2_ surface. As shown in Supplementary Fig. 5, the results suggest the stronger adsorption energy of P atoms on the AuIn_2_ alloy than that on the InP or the In metal. Furthermore, the universality of the method was also explored to examine the as-proposed growth mechanism. DFT calculations of the adsorption energies of Sb atom on various substrates were carried out and the results suggest a stronger adsorption strength of AuIn_2_ alloy compared with that of the In metal and InSb substrates (Supplementary Fig. 6), further proving that the enlarged adsorption strength could enhance the interaction between the substrates and the materials as well as trigger the 2D LBL growth behavior.

### Spectral and structural characterizations of crystals

**Fig. 4:**
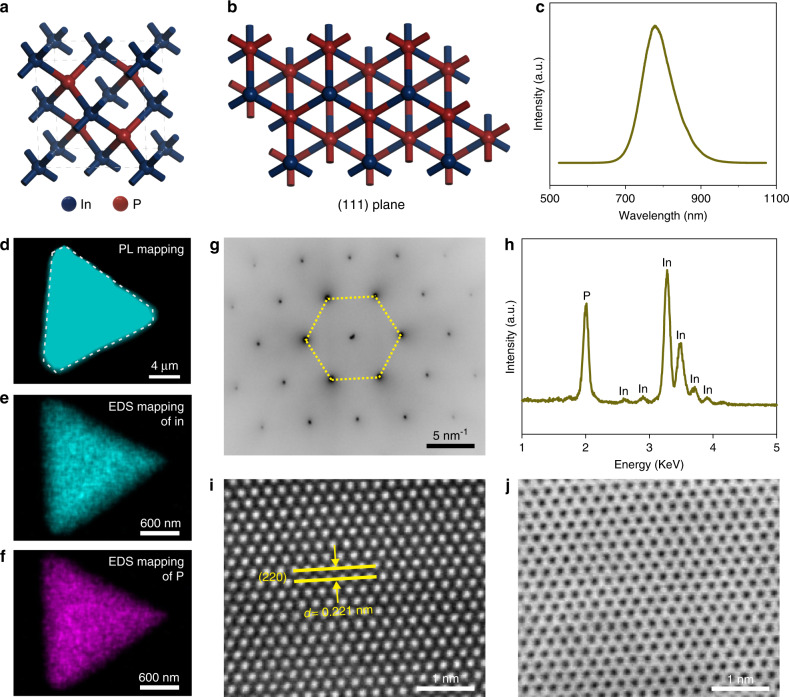
Spectral and structural characterizations of the ultra-thin InP single crystals. **a** Scheme of the typical ZB structure of InP crystal. **b** Scheme for the (111) plane of ZB InP. **c**, **d** The PL spectrum and the corresponding PL mapping of the InP single crystal, respectively. **e**, **f** The EDS mapping of In and P, respectively. **g** The SAED patterns of the InP crystal. **h** The recorded EDS spectrum of InP. **i**, **j** The HAADF–STEM and BF–STEM images of the InP crystal acquired along the [111] zone axis.

For investigating crystal features of ultra-thin InP single crystals, spectral and structural characterizations were performed. The typical ZB structure of InP crystal and its (111) plane are schemed in Fig. 4a and b, respectively, which suggest the inherent covalent interaction in the 3D directions. As the emission wavelength is the most identifiable aspect of a semiconductor with size variation, the photoluminescence (PL) characterization of the ultra-thin InP crystal was implemented to investigate the quantum confinement effect (Fig. 4c). The PL emitting peak of ultra-thin InP single crystal at 778 nm reveals a remarkable blue shift compared with that of the bulk counterparts (919 nm) shown in Supplementary Fig. 7. This easy observation of quantum confinement effect in ultra-thin InP single crystal may result from its large exciton Bohr radius (~10 nm)^28^. In addition, the homogeneous color of the PL mapping (Fig. 4d) image suggests the uniform crystallinity of the InP.

To further confirm the atomic structures of InP single crystals, transmission electron microscopy (TEM) characterization was carried out. The corresponding energy-dispersive X-ray spectroscopic (EDS) mapping images of the crystal suggest a good uniformity of the In and P elements (Fig. 4e, f). The collected selected area electron diffraction (SAED) pattern shows only one set of diffraction spots (Fig. 4g), which confirmed the single-crystal nature of the as-grown InP crystal. The EDS spectrum (Fig. 4h) extracted from the InP crystal indicates that the crystal consists of In and P with a stoichiometric ratio of ~1:1. To further analyze the atomic structure, the high-angle annular dark-field scanning transmission electron microscopy (HAADF–STEM) (Fig. 4i) and the bright-field STEM (BF–STEM) (Fig. 4j) images of the InP single crystal are acquired. The ordered atom sequence of In and P is in good agreement with the structure of (111) plane. The lattice fringes recorded from the STEM image exhibit a distance of 0.221 nm, which corresponds to the (220) interplane spacing of the ZB InP structure. Notably, this acquired interplane spacing is larger than that of the bulk counterpart (0.207 nm, recorded from JPCDS card No. 32–0452), further demonstrating the presence of the increased lattice constant of InP crystal structure. This behavior may be attributed to the change of orbital hybridization induced by the decreased thickness along the *c* axis for the III–V semiconductors^10,11^. Moreover, the time of flight secondary ion mass spectrometry (TOF-SIMS) was employed to exclude the doping of transition elements (Supplementary Fig. 8). Apart from the InP, the InSb single crystals also exhibit high crystallinity and enlarged lattice parameters, as validated by the TEM characterizations (Supplementary Fig. 9).

### Nonlinear optical properties of ultra-thin InP crystals

**Fig. 5:**
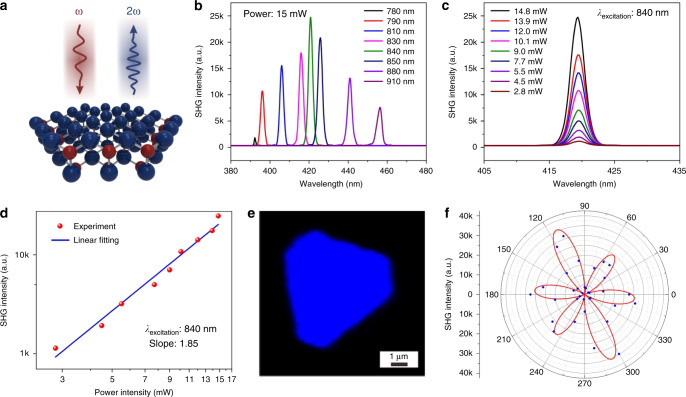
Nonlinear optical properties of the ultra-thin InP single crystal. **a** Schematic of the SHG test. **b** Wavelength-dependent SHG intensity under excitation wavelength from 780 to 910 nm. **c** Excitation power dependence of SHG intensity. **d** The linear fitting of power-dependent SHG intensity in logarithmic coordinates. **e** A SHG mapping of a typical InP single crystal. **f** The polarization-resolved SHG signal of the ultra-thin InP crystal.

Considering that the strongly polarized light emission favoring the nonlinear optics of the III–V semiconductors may occur in the 2D limit according to the predictions^13^, the SHG investigation (Fig. 5a) of the ultra-thin InP single crystals was performed to explore their nonlinear optical properties.

Here, the highest SHG intensity of the ultra-thin InP was recorded at an incident wavelength of 840 nm femtosecond laser and the output power was fixed at 15 mW, as shown in Fig. 5b. To probe the power-dependent SHG intensity of InP, the laser power was tuned from 2.8 to 14.8 mW in 1 s under the excitation wavelength (*λ*_excition_) of 840 nm. As shown in Fig. 5c, with the increase of the power, the SHG intensity exhibits an evident increase at the peak of 420 nm. The relationship between the laser power and SHG intensity is fitted linearly in a double logarithmic coordinate system, leading to a slope value of 1.85 (Fig. 5d), which approaches the theoretical value of 2 extracted from the electric dipole approximation^29^. The direct observation of strong SHG intensity demonstrates the potential of ultra-thin InP crystal in nonlinear optics. The typical SHG mapping presented in Fig. 5e indicates a uniform SHG intensity, further demonstrating its pure phase. Since the polarization-resolved SHG signal is sensitive to lattice symmetry, the angle-dependent SHG intensity of the InP single crystals was measured. The polarization-resolved SHG intensity can be fitted according to the formula of *I*_SHG_ = *I*_0_cos^2^(3*θ*), as shown in Fig. 5f, which exhibits a six-fold anisotropic behavior. It is believed to be induced by the magnified asymmetric structure^30,31^ of the InP with staggered stacking orientation. Notably, the ultra-thin InSb single crystal exhibits a giant SHG response (Supplementary Fig. 10) with the 1 s exciton (1410 nm) at a small laser power (2.05 mW), indicating the extraordinary nonlinear optical properties of ultra-thin InSb.

## Discussion

In summary, we demonstrate that the material-substrate interaction plays a key role in the growth of the ultra-thin III–V crystals because these non-layered materials possess intrinsic covalent bonding in the 3D directions. By constructing the appropriate alloy substrate with enhanced adhesion strength, the growth of III–V crystals can proceed in the 2D LBL growth mode and various representative ultra-thin III–V single crystals can be successfully obtained. We discover that these highly-crystalline ultra-thin III–V systems exhibit remarkable quantum confinement effect and outstanding nonlinear optical properties. The as-achieved breakthrough in the universal synthesis of ultra-thin III–V crystals will greatly promote this material family to win their highlight in the future ultra-thin semiconductor electronics. Our strategy brings forth horizons for realizing the 2D anisotropic growth of the materials, especially for the non-layered systems.

## Methods

### LBL growth process of ultra-thin III–V semiconductors

The growth of ultra-thin III–V single crystals was performed in a quartz tube furnace under ambient pressure. The growth process of ultra-thin InP consists of four steps: (1) Placing the Au wire (~5 mg, 99.999%, China New Metal) and In grain (~6 mg, 99.99%, Alfa Aesar) on the W foil (1 cm × 1 cm, 99.95%, Alfa Aesar) in the center region of the quartz tube; (2) Heating the Au–In–W substrate to 1070 °C under the flow of 300 sccm Ar and 300 sccm H_2_ for 5 min; (3) Cooling the AuIn_2_ alloy substrate to 700 °C at a rate of 30 °C min^–1^ under the flow of Ar and H_2_ to form AuIn_2_ alloy; (4) The AuIn_2_ alloy serving as growth substrate to allow the formation of ultra-thin InP single crystals after heating the P powders (~0.5 mg, 99.999%, Alfa Aesar) at 200 °C for 15–25 min. While in the growth process of ultra-thin InSb, the crystals were synthesized on the AuIn_2_ alloy at 700 °C when heating the Sb powders (~0.5 mg, 99.5%, Alfa Aesar) at 650 °C for 15–20 min. AuGa alloy was fabricated by ~5 mg Au wire and ~1.8 mg Ga grains (99.99%, Alfa Aesar) via heating at 1070 °C for 5 min. The ultra-thin GaP single crystals were grown at 600 °C with the P powders (~0.5 mg) heated at 200 °C for 15–20 min. For the growth process of ultra-thin GaSb single crystals, AuGa alloy was heated at 700 °C when heating the Sb powders (~0.5 mg) at 650 °C for 15–20 min. For the growth process of ultra-thin GaN single crystals, AuGa alloy was heated at 1000 °C when heating the (NH_2_)_2_CO powders (~5 mg, 99.5%, Aladdin) at 200 °C for 5–15 min.

### Transferring process of the ultra-thin crystals

The transferring process consists of five steps: (1) Spin-coating the poly(methyl methacrylate) (PMMA) films onto the AuIn_2_ substrates with the InP or InSb crystals grown on them; (2) Etching out AuIn_2_ substrates with the diluted hydrogen chloride (volume ratio of 1:6) for 6–8 h. Consequently, the PMMA/sample films were then released; (3) Removing the metal ions by rinsing with deionized water; (4) Transferring PMMA/sample films onto the target substrates, e.g., Si/SiO_2_ substrates and Cu grids; (5) Dissolving the PMMA layers with hot acetone for obtaining the samples for the subsequent characterization.

### Characterizations

The XRD characterization was conducted with a Rigaku Miniflex600. Raman and PL spectra were performed with a laser micro-Raman spectrometer (Renishaw in Via, 532 nm excitation wavelength). SEM images were obtained on a ZEISS Merlin Compact SEM. The AFM images were taken with an NT–MDT Ntegra Spectra. The XPS measurements were carried out using a Thermo Scientific, ESCALAB 250Xi. The XPS depth analysis was performed at 3 keV with a spot size of 2 mm × 2 mm and the binding energies were calibrated by referencing the C 1 s peak (284.8 eV). The TEM images were obtained using an aberration-corrected transmission electron microscope (FEI Titan Thermis 60–300) operated at 300 kV. The STEM images were obtained using a probe Cs-corrected transmission electron microscope (FEI Titan ChemiSTEM) operating at 200 kV. The positive ion TOF-SIMS spectra were acquired with an ION TOF ToF SIMS 5-100.

### SHG measurements

The power-dependent SHG measurements of InP crystals were conducted with a Chameleon Ti:Sapphire laser (pulse width 80 fs, repetition frequency 80 MHz). The SHG signal was collected by the WITec alpha 300RS+ Raman system with a ×100 objective (NA = 0.9). The SHG measurements of InSb crystals were conducted with supercontinuum source, SC-PRO-7, YSL. The SHG signal was collected by the Action SpectraPro SP-2300 (300 mm Triple Grating Imaging Spectrometer) with a ×100 objective (NA = 0.7).

### DFT calculations

The DFT calculations of the adsorption energies of atoms were carried out using the CASTEP^32^ module in the Materials Studio software (Bio Accelrys). Perdew–Burke–Ernzerhof (PBE) within the generalized gradient approximation (GGA) function^33^ was used for the treatments of electron exchange and correlation energy. The single atoms of P and Sb were placed in a 20 × 20 × 20 Å^3^ cubic cell for calculating the single point energy values, where the energy cutoffs were set to be 320 eV for the single atoms. The geometry optimization of all the systems was performed with a vacuum space of 20 Å. The self-consistent-field (SCF) convergence criterions were adopted to be smaller than 10^–6^ eV per atom in the calculation. The adsorption energy (*E*_ads_) of the P atom on the AuIn_2_ layer was calculated based on the equation of $E_{{\mathrm{ads}}} = E_{{\mathrm{P}} - {\mathrm{AuIn}}_2} - E_{\mathrm{P}} - E_{{\mathrm{AuIn}}_2}$, where $E_{{\mathrm{P - AuIn}}_2}$ was the energy of P-involved AuIn_2_ system, *E*_P_ was the energy of the P atom and $E_{{\mathrm{AuIn}}_2}$ was the energy of the AuIn_2_ layer. The values of *E*_ads_ of other systems were calculated based on the similar equation. The interactions between InP layers and substrates were calculated by the first-principles method with the Vienna ab initio Simulation Package (VASP)^34,35^. The exchange-correlation interaction is GGA–PBE functional and the projector-augmented wave (PAW) technology is used to describe the core electrons^36^. The kinetic energy cutoff for plane-wave basis is 350 eV. The relaxation of the electronic degrees of freedom is stopped when the total energy difference is below 10^–5^ eV per unit in self-consistent loop, and the structure relaxation is stopped until the max force between atoms is lower than 0.02 eV Å^–1^. The Grimme-D3(BJ) correction^37,38^ is used to describe the interaction between the InP layer and substrates.

## Supplementary information

Supplementary Information

## Data Availability

The data that support the findings of this study are available from the corresponding author upon reasonable request.
