# Supplementary material for: Universal growth of ultra-thin III–V semiconductor single crystals
Source: Nat Commun. 2020 Aug 7;11:3979. doi: 10.1038/s41467-020-17693-5 (PMC7414113; doi:10.1038/s41467-020-17693-5)
Supplement: Supplementary file 1 — Supplementary Information [file 41467_2020_17693_MOESM1_ESM.pdf]

Supplementary Information

Universal growth of ultra-thin III–V semiconductor single crystals

Chen et al

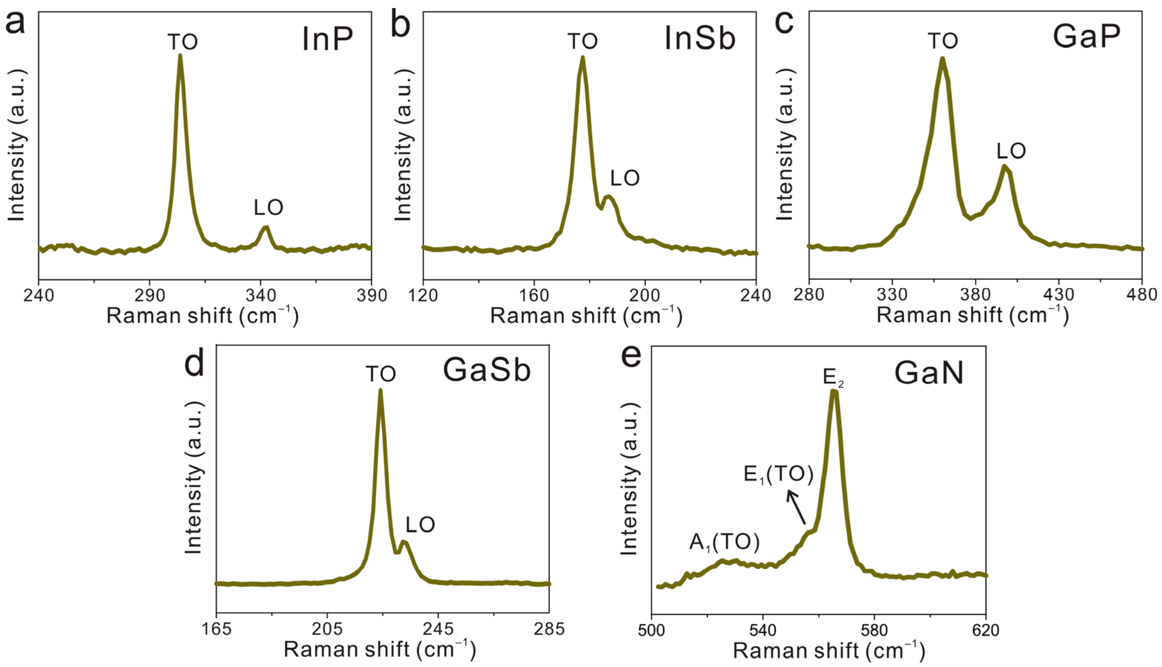

Supplementary Figure 1. a–e, The Raman spectrum of ultra-thin InP, InSb, GaP, GaSb, and GaN single crystals, respectively.

The two Raman peaks located at 360 cm^{-1} and 399 cm^{-1} (Supplementary Fig. 1c) are indexed to the transverse optical (TO) and longitudinal optical (LO) phonon modes of GaP[1], respectively. The two Raman peaks located at 224 cm^{-1} and 233 cm^{-1} (Supplementary Fig. 1d) are indexed to the TO and LO phonon modes of GaSb, respectively[2].

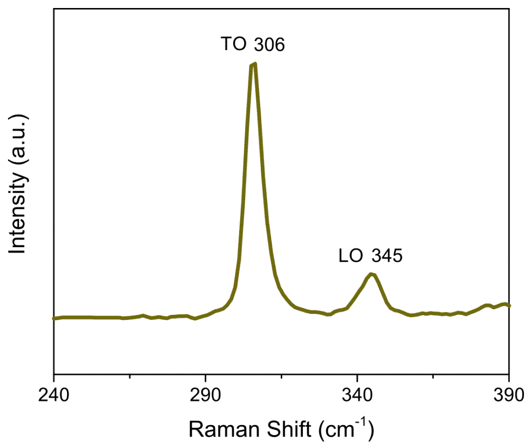

Supplementary Figure 2. Raman spectrum of the bulk InP, where the TO and LO phonon frequencies are located at 306 cm^{-1} and 345 cm^{-1}, respectively.

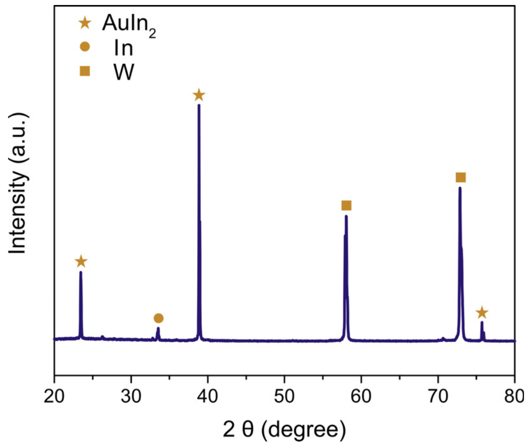

Supplementary Figure 3. X-ray diffraction (XRD) characterization for identifying the formation of $AuIn_2$ alloy.

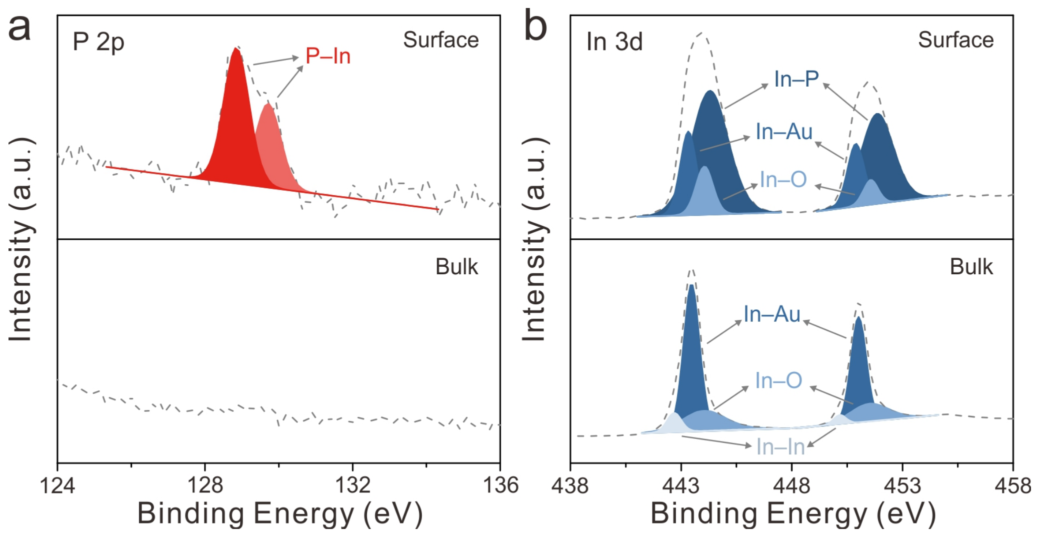

Supplementary Figure 4. a and **b**, The X-ray photoelectron spectroscopy (XPS) depth analysis of $AuIn_2$ substrates after the growth of InP for P 2p and In 3d, respectively.

The XPS depth analyses of the $AuIn_2$ alloy samples after growing ultra-thin InP crystals are presented in Supplementary Fig. 4 and C 1s peak was fixed at 284.8 eV for the calibration of the peak position. For the surface of the substrate, the P 2p spectrum shows two peaks centered at 128.8 and 129.7 eV, which are attributed to P $2p_{3/2}$ and P $2p_{1/2}$ corresponding to the P–In bond[3,4], as seen in the upper panel of Supplementary Fig. 4a). In the case of In 3d spectrum, the two peaks appearing at 444.3 and 451.9 eV are assigned to the In $3d_{5/2}$ and In $3d_{3/2}$ spin-orbit components corresponding to In–P bond[5], as seen in the upper panel of Supplementary Fig. 4b. These results indicate the formation of InP crystals on the surface of the $AuIn_2$ substrate. In addition, the In 3d peaks located at 443.3 and 450.9 eV can be ascribed to the In–Au components[6] and the peaks that located at 444.1 and 451.6 eV can be ascribed to the In–O components[7], which come from the $AuIn_2$ substrate and the substrate oxidation. The chemical compositions of the substrate bulk were also characterized after Ar-ion sputtering. No obvious peak assigned to the P 2p signal corresponding to the P–In bond is observed, as shown in the lower panel of Supplementary Fig. 4a. For In 3d spectra presented in the lower panel of Supplementary Fig. 4b, the two main subpeaks located at 443.5 and 451.0 eV are in line with the In–Au components. The presence of In–O components and In–In components can be observed in the bulk owing to the emerging subpeaks centered at 444.0, 451.5 eV and 442.7, 450.2 eV, respectively.

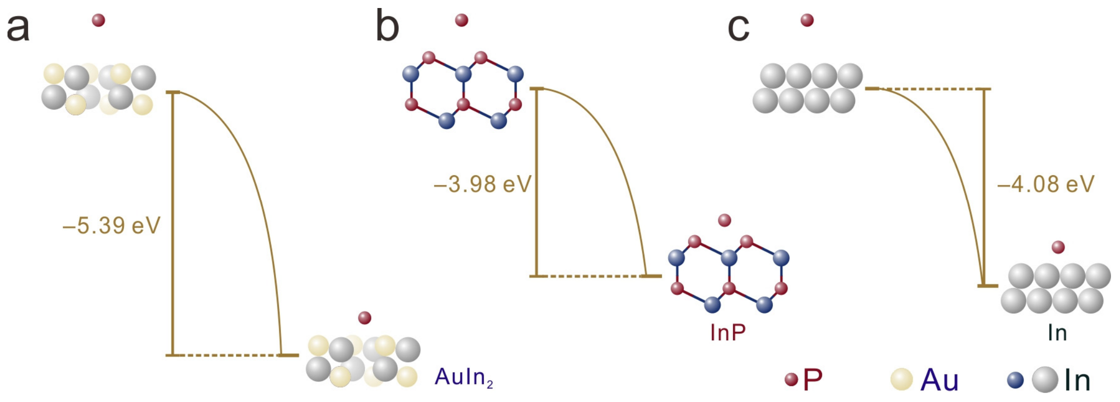

Supplementary Figure 5. a, **b**, and **c**, The adsorption energies of P atoms on the surfaces of $AuIn_2$, InP, and In substrates, respectively.

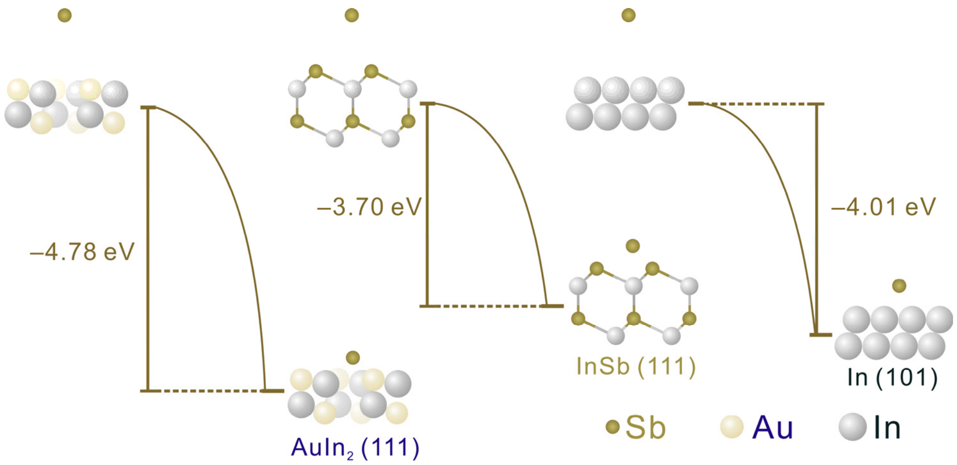

Supplementary Figure 6. DFT calculations of the adsorption energies of Sb atom on various substrates. The adsorption energies on the $AuIn_2$ alloy substrate, InSb substrate, and pure In substrate are –4.78eV, –3.70 eV, and –4.01 eV, respectively.

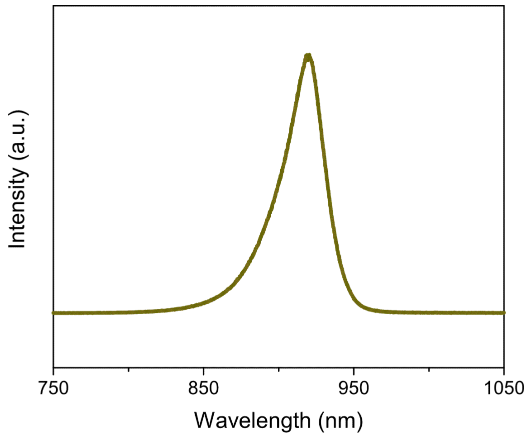

Supplementary Figure 7. Photoluminescence (PL) spectrum of the bulk InP crystal, where the peak is located at 919 nm.

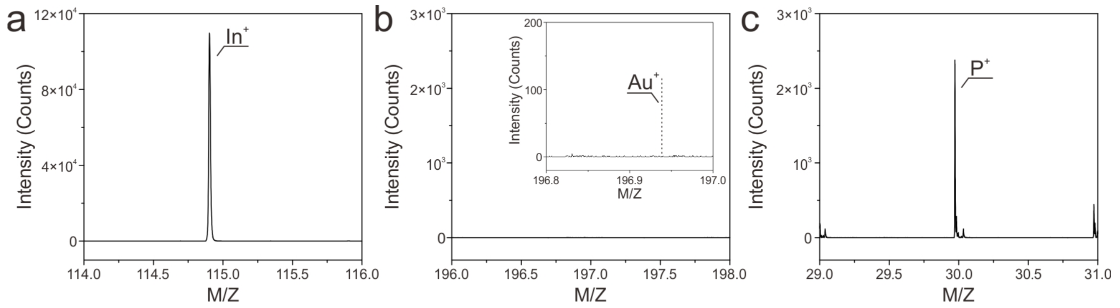

Supplementary Figure 8. a–c, The positive ion time of flight secondary ion mass spectrometry (TOF–SIMS) spectra of the ultra-thin InP sample grown the $AuIn_2$ surface.

For investigating the doping levels of the ultra-thin crystals obtained in the LBL growth process, the TOF–SIMS analysis was employed to explore the compositions of the transferred InP crystals on Si/SiO_2 substrate. Here, the positive ion TOF-SIMS spectra acquired from the sample grown on the $AuIn_2$ surface indicate the obvious peaks assigned to In^+ (Supplementary Fig. 8a) and P^+ (Supplementary Fig. 8c), where the peak assigned to Au^+ is absent (Supplementary Fig. 8b). The results indicate that no obvious transition metal element doping is observed in the ultra-thin InP single crystals.

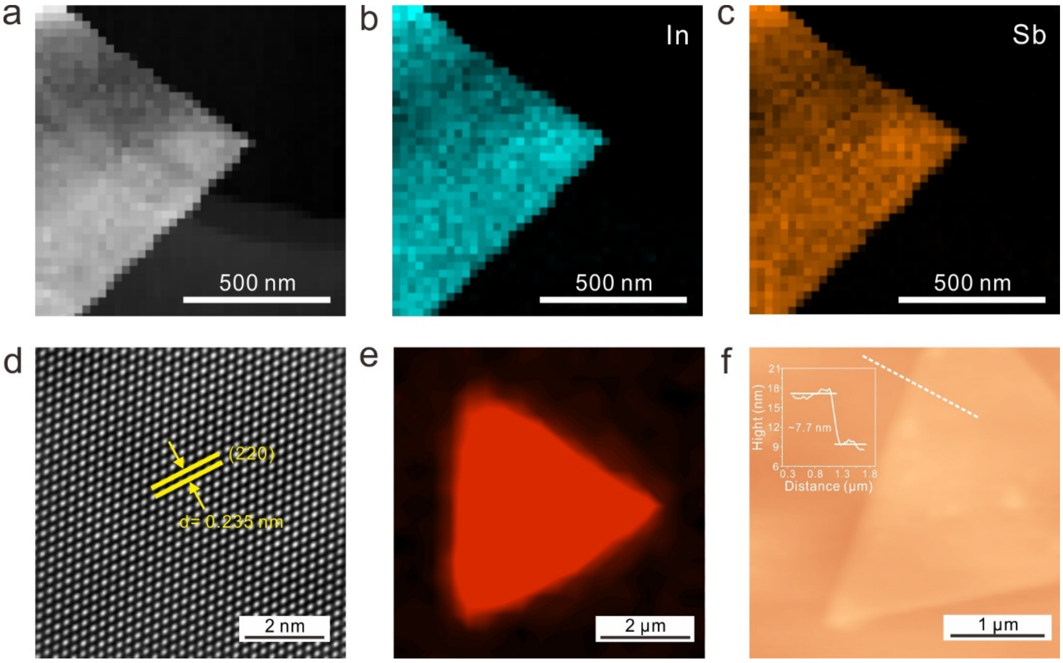

Supplementary Figure 9. Characterizations of the ultra-thin InSb single crystal. a, A low-magnification high-angle annular dark-field scanning transmission electron microscopy (HAADF–STEM) image of the ultra-thin InSb crystal. **b** and **c**, EDS mapping of In and Sb, respectively. **d**, High-resolution transmission electron microscopy (HRTEM) image of the ultra-thin InSb crystal. **e**, Raman mapping of the InSb crystal. **f**, Atomic force microscopy (AFM) image of the InSb crystal. The inset shows the corresponding height profile.

TEM characterizations of the ultra-thin InSb single crystals were carried out for validating their chemical composition and crystal quality. The typical low-magnification HAADF–STEM image of an ultra-thin InSb crystal and the corresponding EDS mapping are presented in Supplementary Fig. 9a–c, which shows a uniform distribution of In and Sb among the crystal. The HRTEM image of the ultra-thin InSb crystal is displayed in Supplementary Fig. 9d, where the well-ordered atomic arrangement of In and Sb atoms without observable defect demonstrates the high crystallinity of it. The recorded distance of 0.235 nm is assigned to the (220) interplane spacing value of the as-grown ultra-thin InSb crystal, which is larger than that of the bulk zinc blende (ZB) phase InSb (0.229 nm recorded from JPCDS card No. 06–0208). The suggested lattice enlargement is believed to be derived from the lattice relaxation of InSb in its 2D limit, which is well demonstrated by the AFM measurement of the crystal with a recorded thickness of ~7.7 nm (Supplementary Fig. 9f). The typical Raman mapping of ultra-thin InSb crystal is presented in Supplementary Fig. 9e, which suggests a uniform crystallinity.

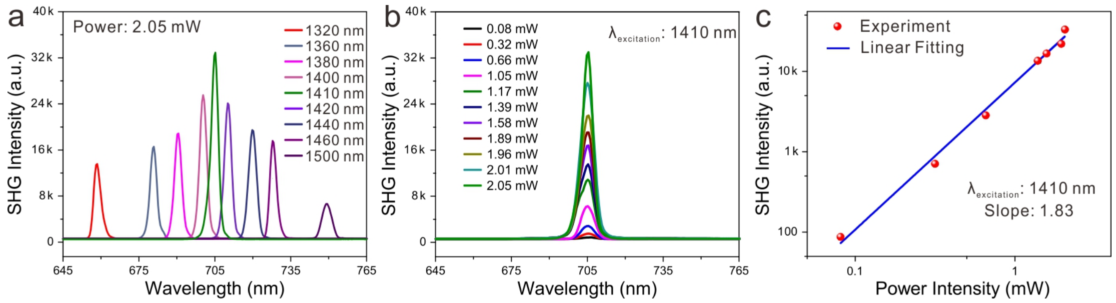

Supplementary Figure 10. Nonlinear optical properties of the ultra-thin InSb single crystals. a, Wavelength-dependent second harmonic generation (SHG) intensity under excitation wavelength from 1320 to 1500 nm. **b**, Excitation power-dependent SHG intensity of the ultra-thin InSb single crystal at the excitation wavelength of 1410 nm. **c**, The linear fitting of power-dependent SHG intensity in logarithmic coordinates.

Supplementary References

1. Wu, Q. *et al.* Synthesis and optical properties of gallium phosphide nanotubes. *J. Phys. Chem. B* **109**, 19719–19722 (2005).

2. Maslar, J., Hurst, W. & Wang, C. A. Raman spectroscopy of n-type and p-type GaSb with multiple excitation wavelengths. *Appl. Spectrosc.* **61**, 1093–1102 (2007).

3. Faur, M., Faur, M., Jayne, D., Goradia, M. & Goradia, C. XPS investigation of anodic oxides grown on p-type InP. *Surf. Interface Anal.* **15**, 641–650 (1990).

4. Nelson, A. J., Frigo, S. & Rosenberg, R. Soft x-ray photoemission characterization of the H_2S exposed surface of p-InP. *J. Appl. Phys.* **71**, 6086–6089 (1992).

5. Kohiki, S., Ohmura, T. & Kusao, K. Appraisal of a new charge correction method in X-ray photoelectron spectroscopy. *J. Electron. Spectrosc. Relat. Phenom.* **31**, 85–90 (1983).

6. Jayne, D. T., Fatemi, N. S. & Weizer, V. G. An x-ray photoelectron spectroscopy study of Au_xIn_y alloys. *J. Vac. Sci. Technol. A* **9**, 1410–1415 (1991).

7. Fan, J. C. & Goodenough, J. B. X-ray photoemission spectroscopy studies of Sn-doped indium-oxide films. *J. Appl. Phys.* **48**, 3524–3531 (1977).
